# Dynamic contrast-enhanced MRI and tumor microenvironment in hepatocellular carcinoma: exploratory analysis of CD3 + TILs, PD-L1, and glypican-3

**DOI:** 10.1186/s41747-026-00834-8

**Published:** 2026-11-03

**Authors:** Daniele Romeo, Jacob Jalil Hassan, Felix Pfister, Anne-Kathrin Höhn, Hans-Michael Tautenhahn, Daniel Seehofer, Uwe Scheuermann, Florian van Bömmel, Timm Denecke, Hans-Jonas Meyer

**Affiliations:** 1 Department of Diagnostic and Interventional Radiology, University of Leipzig, Leipzig, Germany; 2 Department of Biomedicine, Neuroscience and Advanced Diagnostics (BiND), University of Palermo, Palermo, Italy; 3 Department of Pathology, University of Leipzig, Leipzig, Germany; 4 Department of Visceral, Transplant, Thoracic and Vascular Surgery, University of Leipzig, Leipzig, Germany; 5 Division of Hepatology, Department of Medicine II, Leipzig University Medical Center, Leipzig, Germany

**Keywords:** Carcinoma (Hepatocellular), Glypicans, Lymphocytes (Tumor-infiltrating), Magnetic resonance imaging, Tumor microenvironment

## Abstract

**Objectives:**

Dynamic contrast-enhanced (DCE) magnetic resonance imaging (MRI) is the clinical diagnostic standard in hepatocellular carcinoma (HCC), but its association with the tumor microenvironment is not fully characterized. In this study, we investigated correlations between DCE-MRI parameters and immunohistochemistry features of HCC.

**Materials and methods:**

Forty patients, six women and 34 men aged 61.5 ± 10.1 years (mean ± standard deviation) with histologically confirmed HCC were included. DCE-MRI with Gd-EOB-DTPA was performed before surgery, and quantitative parameters were calculated to reflect arterial wash-in, portal venous wash-out and hepatobiliary uptake. Pathological specimens were analyzed for programmed death ligand 1 (PD-L1), glypican-3, CD3+ tumor- and stromal-infiltrating lymphocytes, CD68 and CD34.

**Results:**

CD3+ stromal lymphocytes infiltration was inversely correlated with the arterial wash-in (*r* = -0.47; adjusted *p*-value = 0.025) and several post-arterial parameters. For the other TME components, only exploratory correlations were identified, none of which were statistically significant after adjustment for multiple testing. CD3+ tumoral infiltration lymphocytes with arterial wash-in (*r* = -0.35) and transitional phase relative signal intensity (SI) (*r* = 0.35). Native SI was inversely correlated with PD-L1 (*r* = -0.32). Difference in SI between the transitional and portal phase correlated with glypican-3 expression (*r* = 0.32).

**Conclusion:**

DCE-MRI shows a moderate association with stromal infiltrating lymphocytes (CD3⁺ SIL) in HCC. Associations with other components of the TME, including tumor-infiltrating lymphocytes (CD3⁺ TIL), PD-L1 and Glypican-3 expression, were observed at uncorrected thresholds, but did not remain statistically significant after correction for multiple testing.

**Key Points:**

***Question*** DCE-MRI constitutes the cornerstone of HCC diagnosis. Is quantitative assessment of wash-in, wash-out and hepatobiliary uptake associated with non-invasive characterization of the TME?

***Findings*** CD3⁺ SIL were associated with tumor wash-in and wash-out parameters. Exploratory associations involving CD3⁺ TIL, PD-L1, and glypican-3 lost significance after multiple-testing adjustment.

***Relevance statement*** Quantitative DCE-MRI parameters may provide additional non-invasive information regarding tumor tissue characteristics and the tumor microenvironment of HCC.

**Graphical Abstract:**

**Figure:**
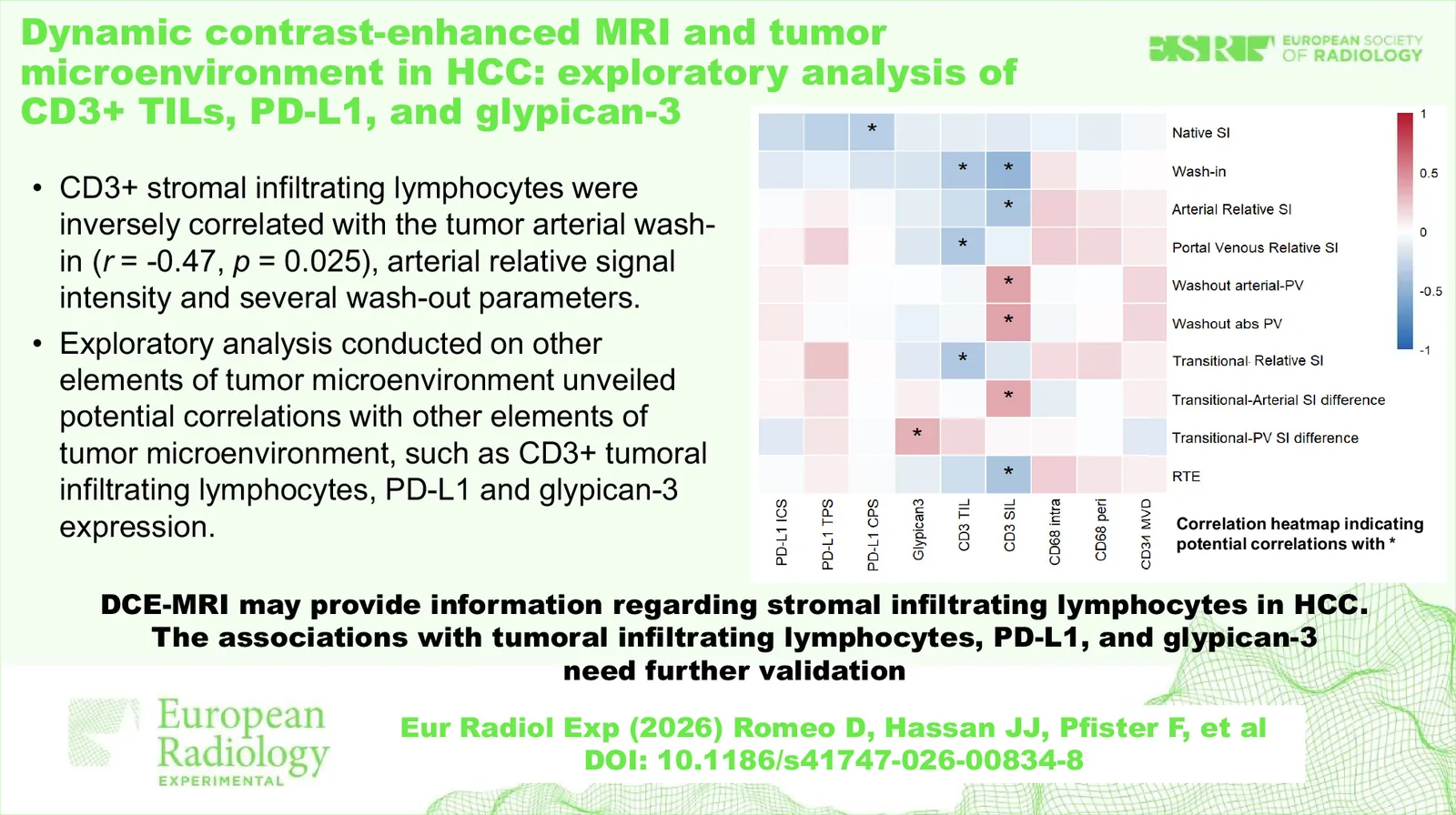

## Introduction

Hepatocellular carcinoma (HCC) is the most common primary liver cancer [1]. In recent decades, although HCC prognosis has shown substantial improvements, overall survival and local recurrence rates remain unsatisfactory, underscoring the need for better tumor characterization to improve pharmacological approaches [2–4].

With the progressive understanding of the relationship between tumor microenvironment (TME) and immunotherapy, new perspectives for HCC therapy are arising. The TME constitutes a crucial key player in HCC response to immunotherapy [2, 4]. However, even though the emerging role of new therapies, such as immune checkpoint inhibitors, is very promising, heterogeneity in treatment responses among patients remains poorly understood, and new studies are needed to elucidate the molecular complexity underlying these phenomena [4, 5].

Notably, it has been demonstrated that tumor contrast media enhancement characteristics are related to HCC biology, reflecting histopathology, including arterial supply and, to some degree, tumor grading [6–9]. Radiomics models have been shown to potentially predict the macrotrabecular massive subtype of HCC [10] and microvascular invasion in post-operative neoadjuvant transarterial chemoembolization (TACE)- treated cohorts [11]. Radiogenomics analyses, correlating radiomics with genomics and transcriptomics, have demonstrated their ability to decipher the heterogeneity of the TME and microvascular invasion in HCC [12], as well as its immune phenotype [13]. However, although promising, they present different challenges in clinical application due to a relatively time-consuming workflow and the need for further standardization [14].

Noninvasive magnetic resonance imaging (MRI)-based identification of HCC histopathological features could offer a highly valuable contribution to optimizing its treatment. At present, biopsy sampling is not routinely performed in all patients [15], underscoring the potential value of imaging-based approaches for more comprehensive tumor characterization. In recent years, emerging evidence has suggested a potential association between “cluster of differentiation” (CD)3⁺ lymphocyte infiltration and dynamic contrast-enhanced (DCE) imaging features, unveiling a correlation between immunoscore and surrounding arterial phase hyperenhancement in HCC [16]. Although these results are primarily derived from computed tomography imaging, DCE-MRI could provide more robust biological evidence.

Therefore, in this study, we explored the possibility of noninvasively quantifying stromal and tumor-infiltrating lymphocytes (TILs), potentially providing new insights into the cellular and molecular composition of the TME. This perspective has gained importance in recent years as the role of CD3⁺ lymphocytes has been investigated, given their association with improved prognosis in patients undergoing immunotherapy [17].

Furthermore, exploratory analyses were conducted on programmed death-ligand 1 (PD-L1) expression, a feature related to HCC outcomes in immunotherapy; glypican-3 expression; CD34 expression as a marker of microvascular invasion; and CD68 expression, which is involved in overall and relapse-free survival in HCC patients [18].

## Materials and methods

This retrospective study was approved by the institutional ethics committee of the University of Leipzig under approval number 159/25-ek. Informed consent was waived due to the retrospective nature of the study. Patients were identified through the clinically used Picture Archiving and Communication System (PACS), and clinical and surgical information was retrieved from digital medical records. The study period ranged from 2013 to 2021. Selection was based on the availability of a radiology-pathology match; therefore, the cohort does not represent a consecutive surgical series. All patients underwent major liver surgery such as liver resection, hemihepatectomy, or hepatectomy.

Patients were included retrospectively in this study if they fulfilled the following inclusion criteria: (1) pre-interventional contrast-enhanced MRI within 10 months before the surgery, (2) lesion size > 5 mm, (3) available pathologic specimens, and (4) histologically proven HCC. Exclusion criteria were imaging compromised by significant metallic and movement artifacts on presurgical MR images. Consequently, nine patients were excluded.

Altogether, 40 patients, six women (15%) and 34 men (85%), aged 61.5 ± 10.1 years (mean ± standard deviation) with histologically confirmed HCC have been included in the present study. All patients were treatment-naïve for the analyzed lesion.

### MRI protocol

In all patients, MRI was performed on a clinical 1.5-T scanner (Aera, Siemens Health Care). The imaging protocol included T2-weighted single-shot and turbo-spin echo sequences with and without fat suppression (repetition time 1,600 ms; echo time 100 ms). DCE scans were obtained after administration of Gd-EOB-DTPA (0.1 mmol/kg body weight, Primovist®, Bayer HealthCare): T1-weighted gradient-echo sequences in the arterial, portal venous, and transitional phase as well as hepatobiliary phase (HBP) 20 min after contrast media application. The sequence was acquired using a breath-hold technique. The parameters of the sequence were as follows: repetition time 4 ms; echo time 2 ms; matrix 172 × 172; field of view 345 × 345 mm^2^; and flip angle 10°. A diffusion-weighted imaging (DWI) sequence was included in the protocol but not analyzed for the current study.

### Image analysis

Images were analyzed within the clinically used institutional Picture Archiving and Communication System (PACS) and evaluated by a trained radiology resident with 2 years of general experience and by a trained radiology resident with 4 years of general experience. Supervision was provided by a consultant radiologist with 10 years of experience in abdominal and oncological radiology in unclear cases. Signal intensity (SI) was measured using a user-defined polygonal region of interest (ROI) placed in the slice with the largest tumor extension, then manually transferred to the other sequences at the same slice and location. The ROI was initially placed in the portal venous phase, and its consistency was confirmed by comparison with the other sequences. Signal intensities were measured in the native phase, late arterial phase (LAP), portal-venous phase (PVP), transitional phase, and HBP 20 min after contrast media injection. All contrast media phase sequences, except the native phase, were standardized to the native SI, as proposed by Öcal et al and implemented in other works [19, 20].

Measured signal intensities were then used to perform the following quantitative assessments.

*Wash-in* was calculated as SI_LAP_ – SI_Native_ according to the work of Collettini et al, where the SI_LAP_ is the signal intensity of the tumor in the LAP, and SI_Native_ is the signal intensity of the tumor in the pre-contrast imaging [21].

*Relative wash-out* for portal venous phase was calculated comparing the LAP SI with portal venous phase images using the formula proposed by Collettini et al and Öcal et al [19, 21] and was calculated as follows: (SI_PVP_ –  SI_LAP_)/SI_LAP._

*Portal venous absolute wash-out* was calculated according to the formula proposed by Choi et al for the evaluation of adrenal metastases from HCC and renal cell carcinoma. [22]. The applied formula is: (SI_PVP_ –  SI_LAP_)/(SI_LAP_ – SI_Native_).

*Differences in SI involving the transitional phase* were calculated as follows: (SI_Ven _– SI_LAP_)/SI_LAP_ when the arterial phase was used as comparison, and as (SI_Ven _– SI_PVP_)/SI_PVP_ when the portal venous phase was used as comparison [19, 21]. To maintain internal coherence, the superior elements of the mathematical equation were inverted. Therefore, increasing SI of the arterial uptake in LAP is characterized by positive values and a decrease in SI is characterized by more negative values.

*Relative tumor enhancement* (RTE) based on the formula proposed by Öcal et al constitutes a measure of the magnitude of hepatobiliary uptake and was calculated as follows: (SI_HBP_-SI_native_/SI_native_) using the HBP [19].

**Fig. 1:**
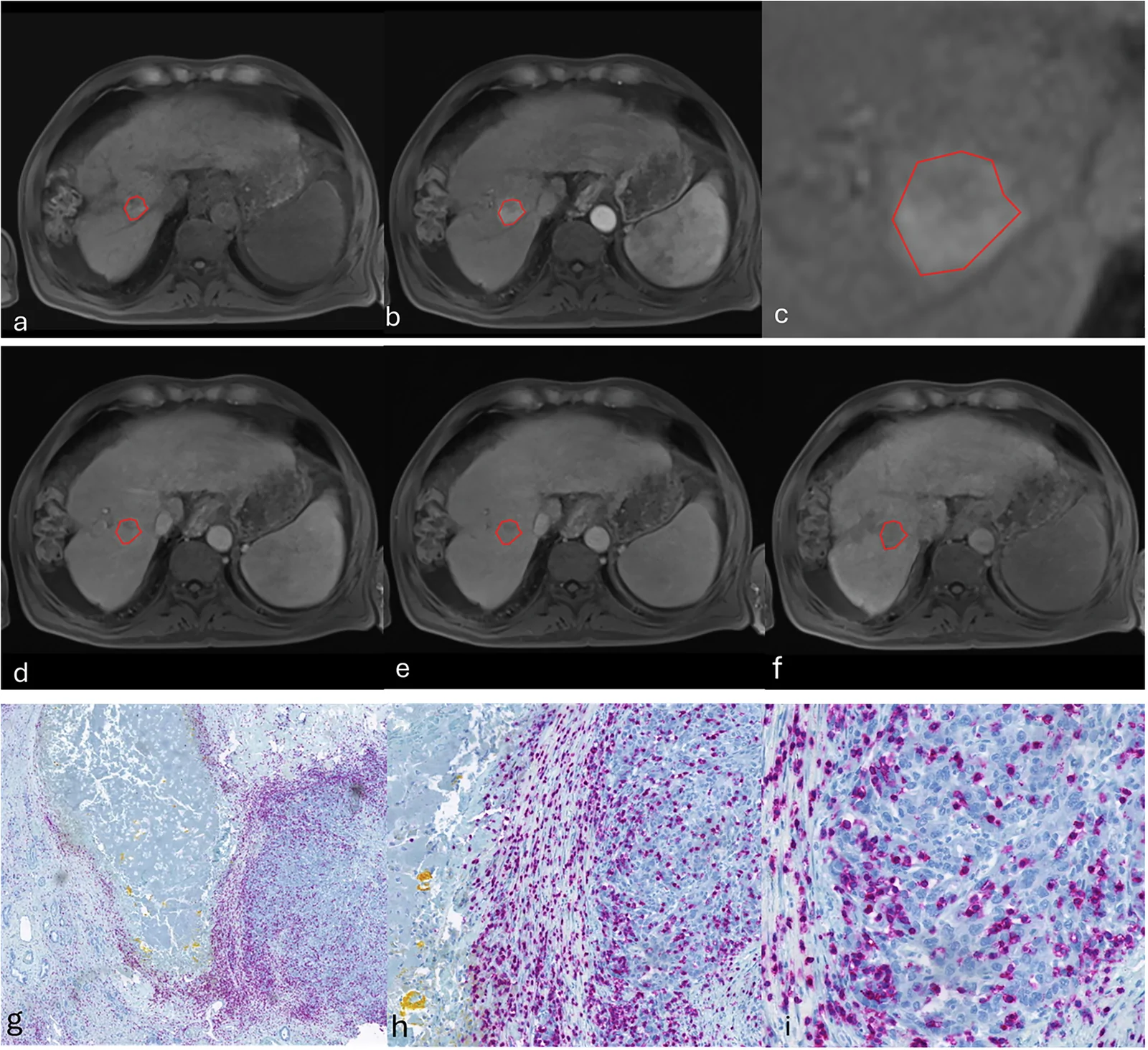
Regions of interest traced on the native (**a**), arterial (**b**), portal-venous (**d**), transitional (**e**), and hepatobiliary (**f**) phase. **c** shows a magnified view of the histologically proven hepatocellular carcinoma. **g**, **h**, and **i** show the lymphocytes CD3⁺ infiltration of the lesion, at 2.0×, 10.0×, and 20.0×, respectively

**Fig. 2:**
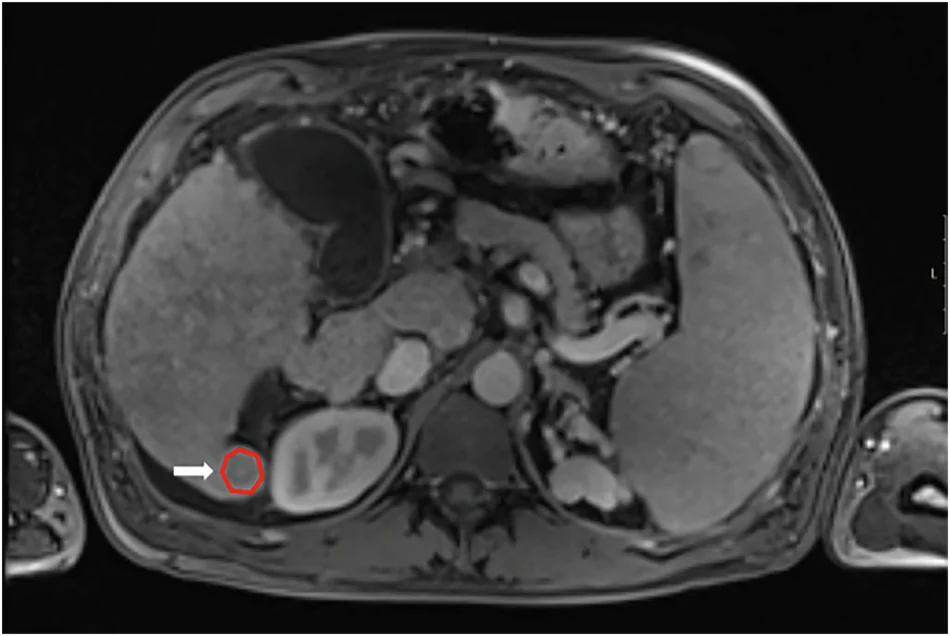
Region of interest placement on portal-venous phase, manually drawn on the slice showing the largest tumor size. The region of interest was placed within the tumor to avoid the partial volume averaging artifact

Figures 1 and 2 provide representative cases of the patient cohort and demonstrate how measurements were performed.

### Histological analysis

Histopathology evaluation was performed by one board-certified pathologist (A.K.H.) with a total of 18 years of general pathology experience, without knowledge of the patients or imaging data. Formalin-fixed, paraffin-embedded tissue serial 3-µm sections were dewaxed in xylol and rehydrated by descending concentrations of ethanol. For each specimen, standard hematoxylin and eosin staining and immunohistochemistry were performed. For antigen detection, we used the automated immunohistochemistry slide-staining system VENTANA BenchMark ULTRA (Roche Diagnostics GmbH), the VENTANA iVIEW DAB Detection Kit, or the ultraView Universal Alkaline Phosphatase Red Detection Kit (Roche Diagnostics GmbH), before counterstaining with Haemalaun solution. All tumors were further investigated with the following immunohistochemistry features: programmed death-ligand 1 (PD-L1, clone 22C3, dilution 1:100, DAKO), glypican-3 (clone IG12, dilution 1:20, DCS), CD3-tumor infiltrating lymphocyte (polyclonal, dilution 1:150, DAKO), CD68⁺ cells (clone PM-M1, dilution 1:100, DAKO) and CD34⁺ microvessel density (clone QBEND10, dilution 1:300, Beckmann Coulter). CD3⁺ cells were evaluated independently in two zones, the intratumoral and stromal areas of the tumor [23].

HCC were categorized per CD3⁺, in the stromal (SIL) and parenchymal areas (TIL), dividing the population into two subgroups: high and low-medium concentration of cells, following the following cut-offs: positive cell clusters with more than 10% of all cells staining positive for CD3⁺ TIL and positive cell clusters with more than 20% of cells staining positive for CD3⁺ SIL [23]. PD-L1 was scored using the combined positive score (CPS), tumor proportion score (TPS), and immune cell score (ICS). For PD-L1 CPS expression, a cutoff level of 1 based on previous studies was used to discriminate the cohort into high and low PD-L1 expression [24, 25]. Microvessel density (CD34⁺) was assessed at 400x magnification with a field area of 0.57 mm^2^.

### Statistical analysis

Statistical analysis and graphics creation were performed using RStudio (version 2024.12.1, R 4.4.3, http://www.r-project.org/, R Foundation for Statistical Computing), GraphPad Prism 8 (GraphPad Software), and SPSS (IBM, version 29.0, SPSS Inc). The collected data were evaluated using descriptive statistics (absolute and relative frequencies). For discrimination analysis, the Mann-Whitney *U*-test was used. For correlation analysis, Spearman’s rank correlation coefficient (*r*) was used to assess associations between the investigated MRI parameters and histopathological features. Benjamini-Hochberg correction was applied to adjust for multiple tests. The results, which were not statistically significant after correction, are reported as explorative. The inter-reader agreement between the two independent observers was evaluated with intraclass coefficients (ICC). In all instances, tests were two-sided, and *p*-values < 0.05 were taken to indicate statistical significance.

## Results

Patient demographics are summarized in Table 1. Liver cirrhosis was present in 35 patients (87.5%), most classified as Child–Pugh A (54.3%). The leading causes were chronic alcohol abuse (57.5%) and hepatitis B (15%). The median time between MRI and histopathology was 94 days (interquartile range: 41–163, min–max range: 3–288). The mean HCC dimension was 3.1 ± 2.3 cm.

**Table 1:** Clinical and demographic characteristics of the included patients

Patient characteristic	Number (%) or mean ± SD
Age (years)	61.5 ± 10.1
Sex	
Male	34 (85.0)
Female	6 (15.0)
Type 2 diabetes mellitus	24 (60.0)
Body mass index (kg/m²)	26.8 ± 3.8
Liver cirrhosis	35 (87.5)
Alcohol use	23 (57.5)
Hepatitis B virus	6 (15)
Hepatitis C virus	5 (12.5)
Other	3 (7.5)
Hepatitis D virus	2 (5)
Child Pugh Class	
A	19 (54.3)
B	13 (37.1)
C	3 (8.6)
Tumor size (cm)	3.1 ± 2.3
Histopathology features	
CD3⁺ TIL	
High	14 (35)
Low	26 (65)
CD3⁺ SIL	
High	21 (52.5)
Low	19 (47.5)
PD-L1 CPS	1.05 ± 4.04
PD-L1 TPS	0.45 ± 1.65
PD-L1 ICS	1.25 ± 4.81
Glypican-3	1.80 ± 0.85
CD 68⁺ peri	1.48 ± 0.51
CD 68⁺ intra	1.3 ± 0.46
CD 34⁺	42.25 ± 10.97

More than one etiology could be present for each patient

*CD* Cluster of Differentiation, *CPS* Combined positive score, *ICS* Immune cell score, *SIL* Stromal infiltrating lymphocytes, TIL Tumor infiltrating lymphocytes, *RTE* Relative tumor enhancement, *SD* Standard deviation, *TPS* Tumor proportion score

The inter-reader agreement was excellent for all investigated MRI parameters, with the best being shown for the native SI measurement (intraclass correlation coefficient 0.988, 95% confidence interval 0.978–0.994).

The median values with interquartile range of calculated DCE-MRI parameters and the corresponding inter-reader agreement are reported in Table 2.

**Table 2:** Inter-reader agreement of the investigated DCE-MRI features

Quantitative assessment	Median	IQR	ICC	95% CI
Native SI	1.335	1.060–1.667	0.988	0.978–0.994
Arterial phase SI	2.11	1.730–2.800	0.964	0.933–0.981
Portal-venous phase SI	2.195	1.803–2.698	0.947	0.899–0.972
Transitional phase SI	2.125	1.713–2.513	0.962	0.927–0.980
Hepatobiliary phase SI	1.96	1.518–2.513	0.968	0.938–0.984
Arterial relative SI	0.577	0.405–0.834	0.965	0.933–0.981
Portal-venous relative SI	0.679	0.400–0.929	0.924	0.857–0.960
Transitional relative SI	0.567	0.346–0.774	0.956	0.917–0.977
Wash-in	0.77	0.583–1.123	0.953	0.911–0.975
Transitional–portal-venous SI difference	-0.057	-0.084 – -0.031	0.652	0.342–0.816
Relative tumor enhancement	0.446	0.256–0.684	0.813	0.641–0.903
Transitional-arterial SI difference	-0.035	-0.118–0.098	0.901	0.813–0.948
Relative portal-venous wash-out	0.0265	-0.08–0.144	0.965	0.933–0.981
Absolute portal-venous wash-out	-0.068	-0.197–0.444	0.693	0.419–0.838

*CI* confidence interval, *DCE-MRI* Dynamic contrast-enhanced magnetic resonance imaging, *ICC* Intraclass correlation coefficient, *IQR* Interquartile range, *SI* signal intensity

**Fig. 3:**
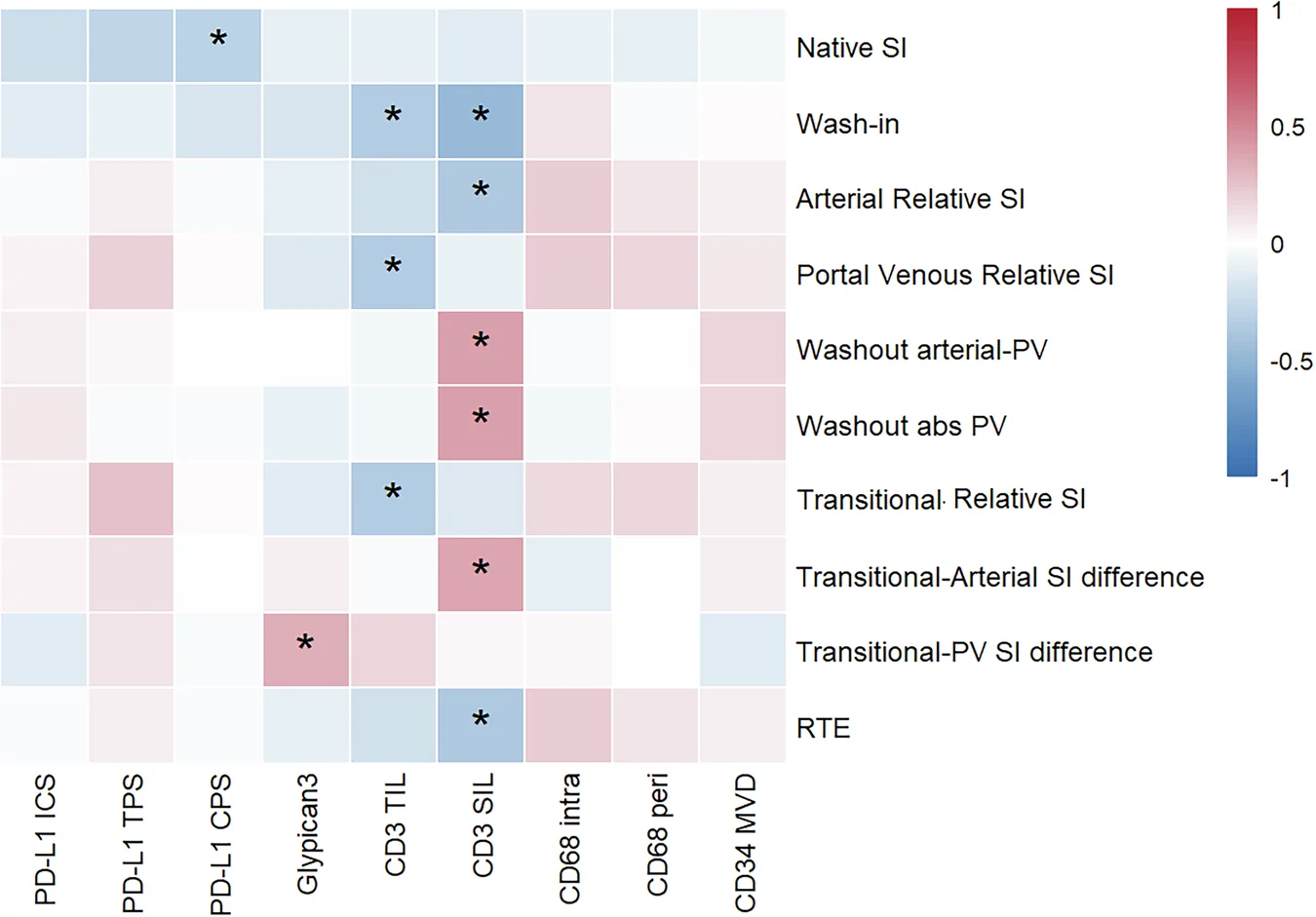
Heatmap showing all the correlations between the radiologically derived quantitative parameters and the histopathological features investigated. Statistical significance (*p* < 0.05) is indicated in the squares by *. CD, Cluster of differentiation; CPS, Combined positive score; ICS, Immune cell score; MVD, Microvessel density; PD-L1, Programmed death-ligand 1; PV, Portal-venous; SI, Signal intensity; SIL, Stromal infiltrating lymphocytes; TIL, Tumor infiltrating lymphocytes; RTE, Relative tumor enhancement; TPS, Tumor proportion score

Total correlation analysis between MRI quantitative features and the underlying histopathology is summarized in a heatmap in Fig. 3, whereby statistically significant correlations are shown in Table 3.

**Table 3:** Overview of the statistically significant correlations between the radiological quantitative assessment and histopathological features

DCE-MRI features	Histopathologic feature	Rho	*p*-value	Adjusted *p*-value
Wash-in	CD3 + SIL	-0.47	0.003	**0.025**
Wash-out absolute portal-venous	CD3 + SIL	0.392	0.013	**0.045**
Wash-out arterial-portal-venous	CD3 + SIL	0.386	0.015	**0.044**
Arterial relative SI	CD3 + SIL	-0.377	0.018	**0.044**
Transitional-arterial SI difference	CD3 + SIL	0.365	0.022	**0.045**
Portal-venous relative SI	CD3 + TIL	-0.357	0.024	0.092
Transitional relative SI	CD3 + TIL	-0.354	0.025	0.092
Wash-in	CD3 + TIL	-0.348	0.028	0.092
Transitional-portal-venous SI difference	Glypican-3	0.325	0.040	0.404
Relative tumor enhancement	CD3 + TIL	-0.327	0.045	0.112
Native SI	PD-L1 CPS	-0.319	0.045	0.451

The reported *p*-values are without Benjamini–Hochberg correction; the adjusted *p*-values are after correction. The significant results after correction are highlighted in bold

*DCE-MRI* Dynamic contrast-enhanced magnetic resonance imaging, *PD-L1* Programmed death-ligand 1, *SI* Signal intensity, *SIL* Stromal infiltrating lymphocytes, *TIL* Tumor infiltrating lymphocytes

The arterial-related parameters showed a negative correlation with CD3⁺ stromal lymphocytes infiltration. The highest correlation was shown from the wash-in of the lesion (*r* = -0.47; *p* = 0.003, after adjustment for multiple testing, *p* = 0.025). Relative arterial phase SI showed a negative correlation (*r* = -0.38; *p* = 0.018; adjusted *p* = 0.044). Several post-arterial parameters showed moderate positive correlations with the CD3⁺ stromal infiltration: absolute portal venous wash-out (*r* = 0.39, *p* = 0.013; adjusted *p* = 0.045), relative portal venous wash-out (*r* = 0.39, *p* = 0.015; adjusted *p* = 0.044), SI difference between the arterial and the transitional phase (*r* = 0.37, *p* = 0.022; adjusted *p* = 0.045).

Wash-in was the only arterial parameter showing correlation with the CD3⁺ tumor infiltration (*r* = -0.35, *p* = 0.028; adjusted *p* = 0.092). Transitional phase relative SI (*r* = -0.35, *p* = 0.025; adjusted *p* = 0.092), portal venous phase relative SI (*r* = -0.36, *p* = 0.024; adjusted *p* = 0.092), and hepatobiliary uptake RTE showed a moderate negative correlation (*r* = -0.33, *p* = 0.045; adjusted *p* = 0.112) with the CD3⁺ TIL further. After adjustment for multiple comparisons, none of these results remained statistically significant.

Only native SI showed a weak negative correlation with one of the PD-L1 parameters (PD-L1 CPS: *r* = -0.32; *p* = 0.045), which did not remain significant after the Benjamini-Hochberg correction (*p* = 0.45).

Regarding glypican-3 expression, a weak positive correlation was identified with the difference in SI calculated between the transitional and the portal venous phase (*r* = 0.33, *p* = 0.04), which did not remain statistically significant after adjustment for multiple testing (*p* = 0.404).

**Fig. 4:**
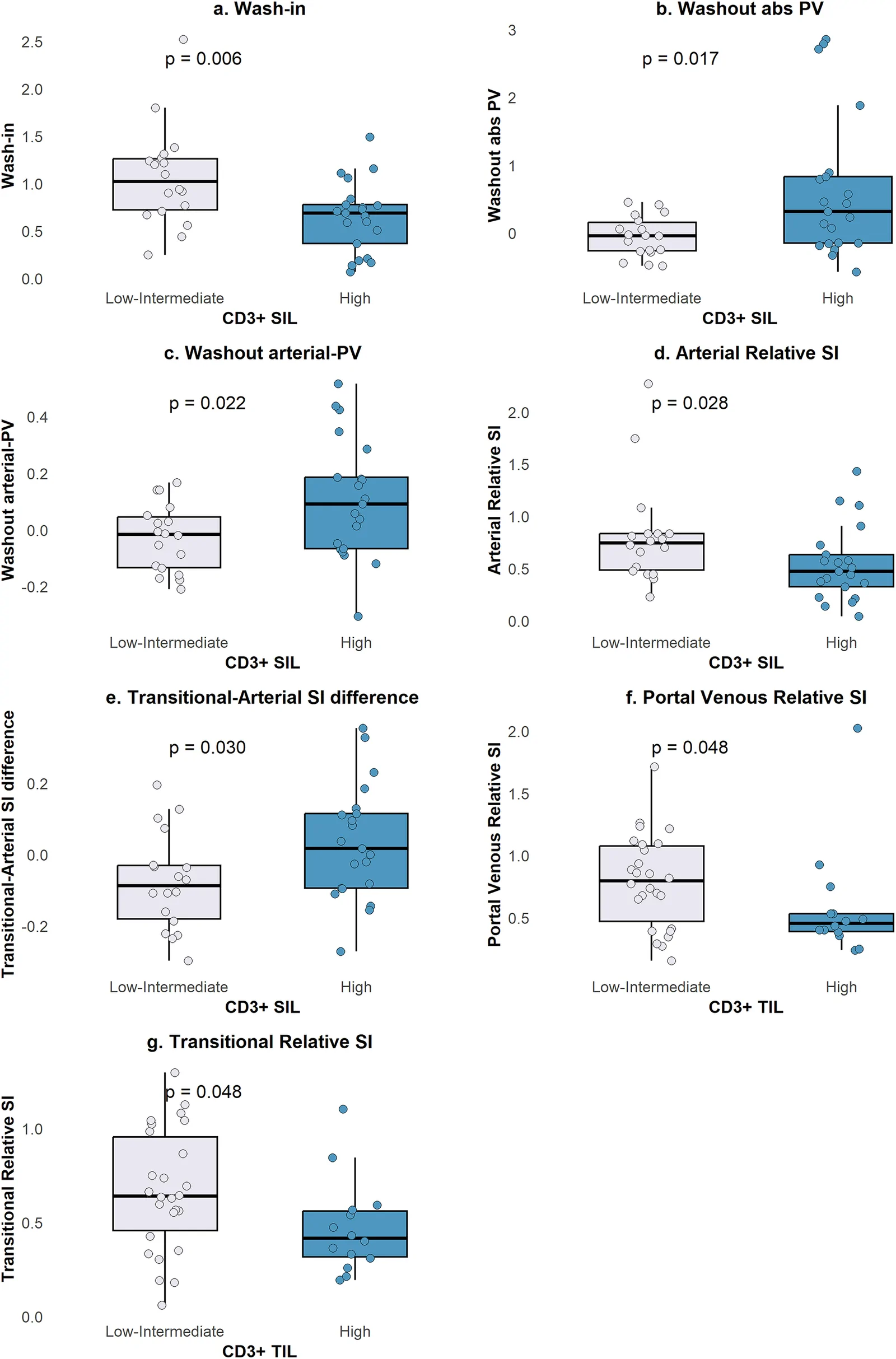
Boxplots showing the statistically significant differences in signal intensity for the quantitative parameters of the object of investigation. **a**, **b**, **c**, **d**, and **e** demonstrate the results for CD3⁺ SILs, while **f**, **g** demonstrate the results of CD3⁺ TILs. *CD* Cluster of differentiation, *SIL,* Stromal infiltrating lymphocytes; *TIL,* Tumor infiltrating lymphocytes

No statistically significant associations were identified for CD68⁺ cells and microvessel density.

Using the aforementioned cutoff, 21 patients were classified as CD3⁺ SIL high, and 19 as CD3⁺ SIL low. Several DCE-MRI parameters discriminated between high and low CD3⁺ stromal infiltration. In short, among the arterial-derived parameters, high and low infiltration were discriminated by wash-in (*p *= 0.006) and the arterial relative SI (*p *= 0.028).

Among the post-arterial-derived parameters, absolute portal venous washout (*p *= 0.017) and portal venous washout (*p *= 0.022), and the difference in SI between the arterial and transitional phases (*p *= 0.03), showed potential to discriminate between the two groups according to high or low stromal CD3+ lymphocytes. Table 4 provides an overview of the comparisons between the groups.

**Table 4:** Discrimination analysis was conducted on the significant radiological quantitative assessment of the CD3+ stromal-infiltrating lymphocytes

DCE-MRI features	High CD3+ stromal infiltration, median (IQR)	Low-medium CD3+ stromal infiltration, median (IQR)	*p-*value
Wash-in	0.690 (0.410)	1.02 (0.538)	**0.006**
Wash-out absolute portal-venous	0.317 (0.979)	-0.038 (0.42)	**0.017**
Wash-out arterial– portal-venous	0.091 (0.252)	-0.016 (0.179)	**0.022**
Arterial relative SI	0.472 (0.306)	0.745 (0.349)	**0.028**
Transitional–arterial SI difference	0.017 (0.210)	-0.087 (0.149)	**0.030**

Statistically significant results are highlighted in bold

*DCE-MRI* Dynamic contrast-enhanced magnetic resonance imaging, *IQR* Interquartile range, *SI* Signal intensity

Using the aforementioned cutoff, 14 patients were classified as CD3⁺ TILs high, while 26 were classified as CD3⁺ TILs low. Only the portal venous relative SI and transitional phase relative SI showed significantly different values, related to the high and medium-low CD3⁺ TILs infiltration (0.45 ± 0.14 [median ± interquartile range] *versus* 0.796 ± 0.606; *p* = 0.048 and 0.42 ± 0.24 *versus* 0.64 ± 0.5; *p* = 0.048). The results are summarized in Table 5. Figure 4 provides the corresponding graphs.

**Table 5:** Discrimination analysis conducted on the significant radiological quantitative assessment of the CD3+ tumor-infiltrating lymphocytes

DCE-MRI features	High CD3+ intratumoral infiltration, median (IQR)	Low-medium CD3+ intratumoral infiltration, median (IQR)	*p*-value
Portal Venous Relative SI	0.455 (0.144)	0.796 (0.606)	**0.048**
Transitional Relative SI	0.416 (0.245)	0.641 (0.497)	**0.048**
Wash-in	0.675 (0.355)	0.870 (0.535)	0.055
Relative tumor enhancement	0.257 (0.433)	0.525 (0.449)	0.071

Statistically significant results are highlighted in bold

*SI* Signal intensity

Using the aforementioned cutoff, six patients were classified as PD-L1 CPS high, while 34 were classified as PD-L1 CPS low. Native SI was higher in the group with lower PD-L1 CPS expression (1.45 ± 0.58 *versus* 0.89 ± 0.47; *p* = 0.012).

No significant differences were identified according to glypican-3 expression (*p* ≥ 0.082).

## Discussion

This study provides an exploratory analysis of quantitative DCE-MRI parameters obtained with Gd-EOB-DTPA and the TME of HCC, including CD3⁺ lymphocyte infiltration, PD-L1 and Glypican-3 expression.

DCE-MRI is a cornerstone of non-invasive HCC diagnosis in clinical practice [26–28]. In at-risk patients, HCC diagnosis relies on evaluation of arterial-phase hyperenhancement and PVP washout [27], as well as ancillary imaging features included in the LI-RADS lexicon, which may help the radiologist adjust the risk category. [27, 29]. However, new discoveries about the composition and roles of the TME would be beneficial for improving immunotherapy outcomes and increasing overall survival among treated patients [30].

The TME comprises cellular components, including adaptive and innate immune cells, fibroblasts, and endothelial cells, as well as non-cellular components such as the extracellular matrix, cytokines, and growth factors [31]. Its immune elements play an important role in the response to immunotherapy and therapies targeting the TME, aiding the implementation of therapeutic strategies, such as immunotherapy or immune checkpoint blockade [31, 32].

In HCC, high concentrations of CD3⁺ lymphocytes in tumor or perivascular regions are associated with a favorable outcome [33, 34]. Ahn et al showed that lymphocyte-rich HCCs have significantly higher overall and relapse-free survival [35]. Another study suggested its potential role in overall and late-phase disease survival after surgical resection [36].

In this study, we independently analyzed the concentration of CD3⁺ T-cells in the stromal and parenchymal portions. Several radiological quantitative features showed a significant correlation with the histopathological evaluation. The stroma is recognized as an important component of the TME, and stromal TILs are involved as key players in cytokine secretion [17]. CD3⁺ stromal infiltration showed a moderate negative correlation with the magnitude of the tumor wash-in in the arterial phase (*r* = -0.47, *p* = 0.003; adjusted *p* = 0.025), while only a weak negative correlation was shown by the arterial relative SI (*r* = -0.377, *p* = 0.018, adjusted *p* = 0.044). Post-arterial phases showed only weak correlations with the CD3⁺ SIL: both relative and absolute portal venous washout showed positive correlations (*r* = 0.386, *p* = 0.015, adjusted *p* = 0.044 and *r* = 0.392, *p* = 0.013, adjusted *p* = 0.045 respectively). Difference in SI between transitional phase and arterial phase also showed a positive weak correlation (*r* = 0.365, *p* = 0.022, adjusted *p* = 0.045). However, these correlations are only moderate to weak.

The analysis involving the CD3⁺ lymphocytes infiltrating the parenchymal portion of the tumor showed that the wash-in (*r* = -0.348, *p* = 0.028), post-arterial measurements of SI in portal venous (*r* = -0.357, *p* = 0.024), transitional (*r* = -0.354, *p* = 0.025) and HBP (*r* = -0.327, *p* = 0.045) were associated with TILs at uncorrected analysis. Although none of the reported correlations remained statistically significant after correction for multiple testing, their consistency across phases may warrant further investigations in larger cohorts to explore the potential contribution of DCE-MRI in tumor lymphocyte infiltration. Among these findings, HBP correlation emphasizes the importance of contrast media uptake as a functional biomarker in HCC, with a high capacity to reflect the underlying histopathology [19]. The HBP hypointensity is associated with the expression of organic anion transporting polypeptide 1B3 (OATP1B3) and is influenced by activation of the Wnt/β-catenin pathway [37, 38].

Despite progress in understanding the biological mechanisms underlying hepatobiliary hypointensity, few studies have explored its connection to the TME. Aoki et al found that CD3⁺ infiltration was significantly higher in immune-inflamed HCC among patients with Wnt/β-catenin mutations (7.49 cells/high-power field microscopy *versus* 91.2 cells/high-power field microscopy, *p* < 0.001). Among the 16 patients who underwent Gd-EOB-DTPA-enhanced MRI, the inflamed class had lower SI in the HBP than the non-inflamed class [39]. Furthermore, DWI analysis was not incorporated in the present study. DWI metrics, particularly apparent diffusion coefficient values, have been associated with tissue cellularity and lymphocyte infiltration in oncological imaging, while in mice they have shown significant correlations with microvessel density and necrosis fraction [40–43]. Therefore, the combination of DCE-MRI parameters and DWI values could provide complementary information regarding lymphocyte infiltration.

Another exploratory finding of this study indicates a potential correlation between native SI and PD-L1 CPS expression in our cohort. PD-L1 is the ligand of PD-1, an immune checkpoint molecule and member of the CD28 family, implicated in reducing T cell activation during the immune response [44]. PD-L1 is expressed in tumor cells, immune cells and antigen-presenting cells, and it plays an important role in the immunosuppressive microenvironment of HCC [44]. Complex crosstalk between tumor and immune cells promotes or inhibits PD-L1 expression in the TME, potentially complicating individualized immunotherapy [45]. In our study, a negative correlation was highlighted between the SI of the native phase and the expression of PD-L1 CPS. This correlation, however, is weak (*r* = -0.319, *p* = 0.045) and did not remain statistically significant after adjustment for multiple testing. These aspects warrant future analyses in larger populations, as tumors with lower expression of PD-L1 CPS showed higher native SI (*p* = 0.012), and PD-L1 CPS constitutes a parameter to predict the efficacy of immunotherapy in several tumor entities [46]. A study by Lee et al demonstrated that high expression of PD-L1 CPS in HCC was associated with improved 12-month overall survival and progression-free survival in a cohort of patients treated with atezolizumab and bevacizumab [25]. A study by Sangro et al shows that overall survival in the entire population differed significantly between PD-L1 high and low patients, using a 1% cut-off regardless of nivolumab treatment (28.1 *versus* 16.6 months; *p* = 0.032) [47].

Glypican-3 is an oncofetal proteoglycan, and its expression is associated with poor prognosis, promoting cell growth and differentiation by activating different cell-signaling pathways [48]. Due to this role, there is an increased interest in glypican-3 as an epitope in HCC targeted therapy and translational research [49].

Tumor-to-liver ratio in the HBP was already identified as quantitative parameter associated with glypican-3 expression in small HCC tumor below 3 cm in size [50], as well as the SMARS scoring system [51] while other studies explored the association between several radiomics parameters and its expression [52, 53].

In our study, the difference in SI between the transitional phase and portal venous phase showed a correlation with glypican-3 expression (*r* = 0.325, *p* = 0.04), although this result did not remain statistically significant after correction (*p* = 0.404) and should, therefore, be considered as exploratory and hypothesis-generating. The measurement could easily be performed by the radiologist in a clinical setting and help with glypican-3 assessment in HCC. The correlation is, in any case, modest, and the lack of confirmation in the discriminatory analysis for this parameter should be accounted as a limitation in our assessment. However, the reported correlations of CD3⁺ TILs, PD-L1 and glypican-3 did not remain significant after applying the Benjamini-Hochberg correction, indicating that these results could only be considered in an exploratory fashion.

Several limitations of the present analysis should be taken into consideration. First, this study is a retrospective single-center study with a relatively small sample size, which limits the statistical power, potentially introducing selection bias. To overcome potential reader bias, imaging and histopathology assessment were performed blinded to each other. The inter-reader agreement of the measured parameters was good to excellent, demonstrating potential efficacy in clinical routine. Furthermore, the median time between imaging and intervention needs to be acknowledged as a limitation, as it could strongly influence the relationship between imaging and TME, representing a meaningful source of uncertainty in the interpretation of imaging-histopathological correlations. Second, only curatively treated HCC lesions were investigated in the current analysis, as histopathological evaluation was only available for these patients. This hinders possible translation for our identified associations to more advanced HCC stages, for which the identified associations seem more clinically relevant. The current patient sample was not treated with immunotherapy, which seems to be the most promising patient cohort for the identified associations in the present study. Third, most of the correlations found are weak-to-moderate. Although this could reflect the elusive relation between imaging and TME composition, it underscores the need for larger, multicentric studies. In the current cohort, survival data were not available, emphasizing the need for larger studies with available survival endpoints to validate the findings before clinical application. Furthermore, the present analysis does not include characterization of CD3⁺ lymphocyte subpopulations, their functional states and exhaustion, as well as their spatial distribution, which may limit the clinical applicability of the findings. Accordingly, the results should be interpreted as exploratory. Additionally, a single slice ROI approach was used for this analysis. Although the methodological choice was applied due to the high feasibility and reproducibility in a clinical setting without requiring the use of an external software, it constitutes an important limitation of this analysis, as it may hinder the possibility of capturing the HCC's high heterogeneity. Further studies with a volumetric approach, radiomics or deep learning-based models are therefore required, as they are more able to capture the whole tumor characteristics. An external validation for our current results is therefore strictly necessary to improve the imaging guidance of the treatment in the future and to define potential cut-off values that potentially discriminate high from low CD3⁺ infiltrating tumors. Last, the use of Gd-EOB-DTPA restricts the generalizability of the analysis, as the signal intensities in the dynamic post-contrast phases are highly dependent on its pharmacological properties. Further studies using extracellular contrast agents may be necessary to unravel other aspects of the correlations between DCE-MRI and TME.

## Conclusion

In conclusion, parameters derived from dynamic-contrast-enhanced MRI are moderately associated with stromal lymphocyte infiltration in HCC. Associations with other components of the TME, including tumor-infiltrating lymphocytes, PD-L1 expression and glypican-3 expression, were observed at uncorrected thresholds, but did not remain statistically significant after correction for multiple testing and should therefore be considered hypothesis-generating. Notably, different parameters from the contrast media phases may be associated with distinct features of the TME. It remains unclear to what extent imaging can predict the different tumor-micromilieu aspects.

## Data Availability

The anonymized patient data can be provided by the corresponding author upon reasonable request.

## Abbreviations

CD: Cluster of differentiation
CPS: Combined positive score
DCE: Dynamic contrast-enhanced
DWI: Diffusion-weighted imaging
HCC: Hepatocellular carcinoma
HBP: Hepatobiliary phase
LAP: Late arterial phase
MRI: Magnetic resonance imaging
PVP: Portal-venous phase
PD-L1: Programmed death-ligand 1
ROI: Region of interest
RTE: Relative tumor enhancement
SI: Signal intensity
SIL: Stromal infiltrating lymphocytes
TIL: Tumor infiltrating lymphocytes
TME: Tumor microenvironment
TPS: Tumor proportion score
